# Knowledge and perceptions of monkeypox among university students in Riyadh, Saudi Arabia: implications for public health preparedness

**DOI:** 10.3389/fpubh.2026.1717967

**Published:** 2026-04-10

**Authors:** Rasha Assad Assiri, Lashin S. Ali, Dana Kh. Alsharayiah, Rasha Mokhtar Elnagar, Mamdouh Eldesoqui, Wedad Tarik, Bayan F. Alsaleh, Lyan Alotaibi, Nawal S. Gouda, Alaa Abdelaty

**Affiliations:** 1 Department of Basic Medical Sciences, College of Medicine, Princess Nourah Bint Abdulrahman University, Riyadh, Saudi Arabia; 2 Department of Basic Medical Sciences, Faculty of Dentistry, Al-Ahliyya Amman University, Amman, Jordan; 3 Department of Basic Medical Sciences, College of Medicine, AlMaarefa University, Riyadh, Saudi Arabia; 4 Research Center, Deanship of Scientific Research and Post-Graduate Studies, AlMaarefa University, Riyadh, Saudi Arabia; 5 Department of Medical Microbiology and Immunology, Faculty of Medicine, Mansoura University, Mansoura, Egypt; 6 College of Medicine, AlMaarefa University, Riyadh, Saudi Arabia; 7 Microbiology Department, Faculty of Medicine, Northern Border University, Arar, Saudi Arabia; 8 Mansoura Manchester Medical Program, Faculty of Medicine, Mansoura University, Mansoura, Egypt

**Keywords:** Mpox, university students, awareness, attitude, vaccine, Saudi Arabia, public health preparedness

## Abstract

**Introduction:**

The 2022 multi-country outbreak of Mpox highlighted its emergence as a global public health concern. However, evidence regarding Mpox awareness and perceptions among university students in the Middle East remains limited, creating an important gap for targeted health education and outbreak preparedness. This study aimed to assess knowledge, perceptions, and attitudes toward Mpox disease, vaccination, and preventive practices among undergraduate students in Riyadh, Saudi Arabia.

**Methods:**

A cross-sectional survey was conducted between October 2024 and March 2025 among 810 undergraduate students from multiple colleges in Riyadh utilizing convenience sampling and a structured questionnaire. Medical students comprised 72.6% of the sample. Data were analyzed using descriptive statistics, chi-square tests, and multivariable logistic regression. Statistical significance was set at *p* ≤ 0.05.

**Results:**

Overall awareness of Mpox was high; however, important knowledge gaps were identified regarding transmission routes, vaccine availability, and treatment options. While most students had heard of Mpox, uncertainty remained regarding specific transmission pathways and clinical features. The internet and social media were the most reported sources of information, whereas higher knowledge levels were significantly associated with exposure to television/news and public health awareness campaigns. Multivariable logistic regression identified female sex (OR = 1.42), age ≥25 years (OR = 1.91), enrollment in medical programs (OR = 1.68), and exposure to television/news (OR = 1.95) or public health campaigns (OR = 2.28) as significant predictors of moderate-to-good Mpox knowledge. Preventive attitudes were generally positive, with moderate willingness to receive vaccination and stronger support for mandatory vaccination among healthcare workers than the general population.

**Conclusion:**

University students demonstrated high general awareness of Mpox but limited depth of knowledge in key areas related to transmission, vaccination, and treatment. Targeted health education strategies are therefore needed, particularly for non-medical students, male students, and younger age groups. Public health communication should combine the wide reach of digital platforms with credible messaging through traditional media and structured awareness campaigns to improve knowledge and preparedness for emerging infectious diseases.

## 1 Introduction

Mpox is a zoonotic infectious disease caused by the *Monkeypox virus* (MPXV), historically endemic to Central and West Africa. In recent years, its spread to non-endemic regions, including Europe, North America, and Australia, has raised substantial global public health concern due to its evolving epidemiology and potential for international outbreaks. The 2022 multi-country outbreak affected six continents and resulted in tens of thousands of confirmed cases (1). This event highlighted the complex interplay between human behavior, animal reservoirs, and environmental change. Increased susceptibility to Mpox may be partly attributed to the discontinuation of smallpox vaccination in 1980, which had previously provided cross-protection against other Orthopoxviruses, including MPXV (2).

The MPXV, closely related to the variola virus, typically causes fever, malaise, and a characteristic vesiculopustular rash, with lymphadenopathy serving as a key distinguishing clinical feature. Although Mpox was traditionally associated with zoonotic transmission, particularly from rodents, recent outbreaks have demonstrated sustained human-to-human transmission. This shift has prompted a reassessment of its transmission dynamics (3). Transmission occurs through direct contact with skin lesions, bodily fluids, respiratory secretions, and potentially contaminated surfaces or objects. Vertical transmission has also been reported (4). Although Mpox is not classified as a sexually transmitted infection, the 2022 outbreak identified a high proportion of cases among men who have sex with men, suggesting that close physical and sexual contact may facilitate transmission under certain conditions (5). These observations underscore the virus’s ability to exploit new transmission contexts, increasing its relevance as an international public health threat.

Mortality rates in endemic African settings range from approximately 1 to 10%, depending on viral clade and access to healthcare. The West African clade is generally associated with milder disease, whereas the Congo Basin clade demonstrates greater virulence. The outbreak has also highlighted challenges in diagnostic capacity, particularly in low-resource settings, underscoring the need for clade-specific surveillance (6).

Current response measures focus on enhanced surveillance, molecular–based diagnostics, isolation of confirmed cases, and supportive clinical care. Although no Mpox-specific treatment is formally approved, antivirals such as tecovirimat and cidofovir that are originally developed for smallpox have been deployed under emergency use protocols (7). Vaccination remains a cornerstone of prevention, with both pre- and post-exposure prophylaxis, including ring vaccination, recommended for high-risk populations (8). In parallel, effective risk communication, public awareness, and stigma-sensitive messaging are essential to counter misinformation and support outbreak control.

University students represent a critical population for assessing Mpox-related knowledge and perceptions. This group is characterized by high social interaction, mobility, and frequent engagement with digital information platforms, which may influence both exposure risk and the spread of health information during infectious disease outbreaks. Moreover, students in health-related disciplines may serve as future healthcare providers and public health advocates, while non-medical students reflect broader community awareness. Their knowledge, attitudes, and behaviors can therefore influence not only their personal risk but also the health practices of families and communities. Understanding knowledge gaps, attitudes toward vaccination, and preventive behaviors within this population can inform targeted educational interventions and strengthen outbreak preparedness strategies.

Therefore, this study aimed to assess university students’ knowledge, awareness, and perceptions of Mpox, with particular emphasis on transmission routes, clinical features, preventive practices, vaccine acceptance, and attitudes toward outbreak containment following the recent global outbreak.

## 2 Methods

### 2.1 Study design, setting and participants

This descriptive cross-sectional study was conducted among undergraduate university students in Riyadh, Saudi Arabia, between October 2024 and March 2025. A total of 810 students, aged 18 years and above, voluntarily participated. Participants were recruited from various colleges and academic years using a convenience sampling approach, which was considered pragmatic for online student recruitment through social media and institutional emails. This method allowed rapid access to a diverse sample, although it may limit the generalizability of the findings.

### 2.2 Study sample size

The minimum required sample size was calculated using Daniel’s formula (9): $n=(Z^{2}\times p\times (1-p))/d^{2}$ , where Z = 1.96 for a 95% confidence interval, p = expected prevalence (0.5), and d = margin of error (0.05). The estimated sample size was 385 participants. To account for a potential 20% non-response rate, the adjusted sample size was 462 participants. A total of 810 fully completed questionnaires were collected, exceeding the minimum requirement and ensuring adequate statistical power.

### 2.3 Data collection tool

Data were collected using a structured, self-administered questionnaire developed based on the latest guidelines from the World Health Organization (WHO) and the Center for Disease Control and Prevention (CDC), as well as evidence from prior studies. The questionnaire was created using Google Forms and disseminated electronically via social media platforms (WhatsApp, Twitter) and institutional email networks. Only fully completed questionnaires were included in the final analysis; incomplete responses were excluded. Before accessing the questionnaire, participants were provided with a study overview, including its objectives, significance, and voluntary nature. Electronic informed consent was obtained, and confidentiality was assured. Participation was entirely voluntary, and participants could withdraw at any time without consequence. The questionnaire was pilot-tested with a small group of students to ensure clarity and comprehension and was available in both Arabic and English.

### 2.4 Questionnaire structure

The questionnaire consisted of six sections: (i) Sociodemographic Information—age, gender, college, and level of education; (ii) General Knowledge—awareness of Mpox and prior exposure to related information before the outbreak; (iii) Modes of Transmission—understanding of disease spread through physical contact, respiratory droplets, and contaminated objects; (iv) Clinical Symptoms—knowledge of symptoms such as fever, headache, lymphadenopathy, and skin rashes; (v) Risk perception and concerns—participants’ level of fear, perceived risk of a potential global outbreak, and self-reported precautionary behaviors; (vi) Vaccination Acceptance—willingness to receive the vaccine and attitudes toward mandatory vaccination.

Correct responses in sections ii–iv were scored as 1, while incorrect or “do not know” responses were scored 0. The total knowledge score ranged from 0 to 20. Knowledge levels were classified as: poor (0–9 points), moderate (10–14 points), and good (15–20 points). This classification was used as the outcome variable in regression analyses to identify predictors of higher Mpox knowledge. As this was an exploratory study, formal reliability testing of the knowledge items (e.g., Cronbach’s alpha) was not conducted. Attitudes and perceptions in sections v and vi were measured using a five-point Likert scale (strongly agree = 1 to strongly disagree = 5). For statistical analysis, responses were coded numerically. Depending on the analysis, Likert data were treated as ordinal variables or grouped into categories to examine associations with demographic factors and knowledge scores. In descriptive tables, frequencies are presented as raw responses for clarity and interpretability. The questionnaire included both multiple-choice and closed-ended questions to ensure consistency and reliability of responses.

### 2.5 Statistical analysis

Data collected via google forms were exported to Microsoft Excel for initial cleaning and then imported into SPSS version 23 (IBM Corp., Armonk, NY, United States) for analysis. Descriptive statistics, including frequencies and percentages, were used to summarize participants’ sociodemographic characteristics, knowledge, perceptions, and preventive practices. Chi-square (χ^2^) tests were conducted to examine associations between categorical variables. Spearman’s rank-order correlation was applied to assess relationships between students’ perceptions, attitudes, and preventive behaviors regarding Mpox. Multivariable logistic regression was performed to identify independent predictors of good/moderate Mpox knowledge, with results reported as odds ratios (ORs) and 95% confidence intervals (CIs). A *p*-value ≤ 0.05 was considered statistically significant.

### 2.6 Ethical considerations

This study was conducted in accordance with the ethical principles outlined in the Declaration of Helsinki. Ethical approval was obtained from the Institutional Review Board (IRB) of Al-Maarefa University, Riyadh, Saudi Arabia (Approval No.: IRB24-088). Electronic informed consent was obtained from all participants before they completed the online questionnaire. Participation was voluntary, responses were anonymous, and strict measures were taken to ensure data confidentiality throughout the study.

## 3 Results

### 3.1 Demographic characteristics of the study participants

A total of 810 undergraduate students participated, with 62.2% females and 77.8% Saudi nationals. Most participants were aged 18–20 years (56.3%) and enrolled in the College of Medicine (72.6%). Nearly half were in their second year, while senior students (fourth and fifth years) comprised a smaller proportion. Detailed sociodemographic characteristics are presented in Table 1.

**Table 1:** Sociodemographic characteristics of the study participants (*n* = 810).

Variable	Category	No.	%
Gender	Male	306	37.8
	Female	504	62.2
Age (years)	18–20	456	56.3
	21–25	303	37.4
	> 25	51	6.3
Nationality	Saudi	630	77.8
	Non- Saudi	180	22.2
College	Medicine	588	72.6
	Pharmacy	48	5.9
	Applied Medical Sciences	54	6.7
	Engineering	12	1.5
	Education	33	4.1
	Business Administration	21	2.6
	Computer Science and Information System	18	2.2
	Dentistry	18	2.2
	Nursing	18	2.2
Year of study	First	163	20.1
	Second	368	45.4
	Third	183	22.6
	Fourth	81	10.0
	Fifth	15	1.9

No., number of participants; %, percentage of total participants.

### 3.2 Mpox knowledge and awareness

Overall, 85.9% of participants had heard of Mpox, and 58.5% were aware of the WHO’s 2022 public health emergency declaration (Table 2). Basic awareness of disease susceptibility and severity was moderate: 53.7% recognized general susceptibility and 69.6% perceived Mpox as dangerous. Knowledge of transmission routes varied: 70.4% identified human contact, 67.4% animal contact, 48.9% fomite transmission, 45.2% sexual contact, and 33.0% viral persistence on surfaces.

**Table 2:** Participants’ knowledge and perceptions regarding Mpox disease (*n* = 810).

Questions	Yes n (%)	No n (%)	I do not know n (%)
Have you ever heard of monkeypox?	696 (85.9)	114 (14.1)	─
Have you ever heard that the WHO declared the global monkeypox outbreak a public health emergency of international concern on July 23, 2022?	474 (58.5)	336 (41.5)	─
People are generally susceptible to monkeypox	435 (53.7)	75 (9.3)	300 (37.0)
Monkeypox is a dangerous infectious disease	564 (69.6)	36 (4.4)	210 (26)
Monkeypox virus can be transmitted through physical contact with other humans	570 (70.4)	33 (4.1)	207 (25.5)
Monkeypox Virus can be transmitted through physical contact with infected animals	546 (67.4)	15 (1.9)	249 (30.7)
Monkeypox virus can be transmitted through sexual intercourse	366 (45.2)	51 (6.3)	393 (48.5)
Monkeypox virus can be transmitted through sharing contaminated sheets, clothes or needles	396 (48.9)	51 (6.3)	363 (44.8)
Monkeypox can survive for several days on contaminated surfaces	267 (33)	60 (7.4)	483 (59.6)
Do you think monkeypox affects daily activity?	363 (44.8)	303 (37.4)	144 (17.8)
There are currently no specific treatments for monkeypox	234 (28.9)	93 (11.5)	483 (59.6)
There is a vaccine that protects against monkeypox	243 (30)	99 (12.2)	468 (57.8)
Do you expect monkeypox to cause a pandemic like COVID-19?	255 (31.5)	306 (37.8)	249 (30.7)

N, number of participants; %, percentage of total participants.

**Figure 1:**
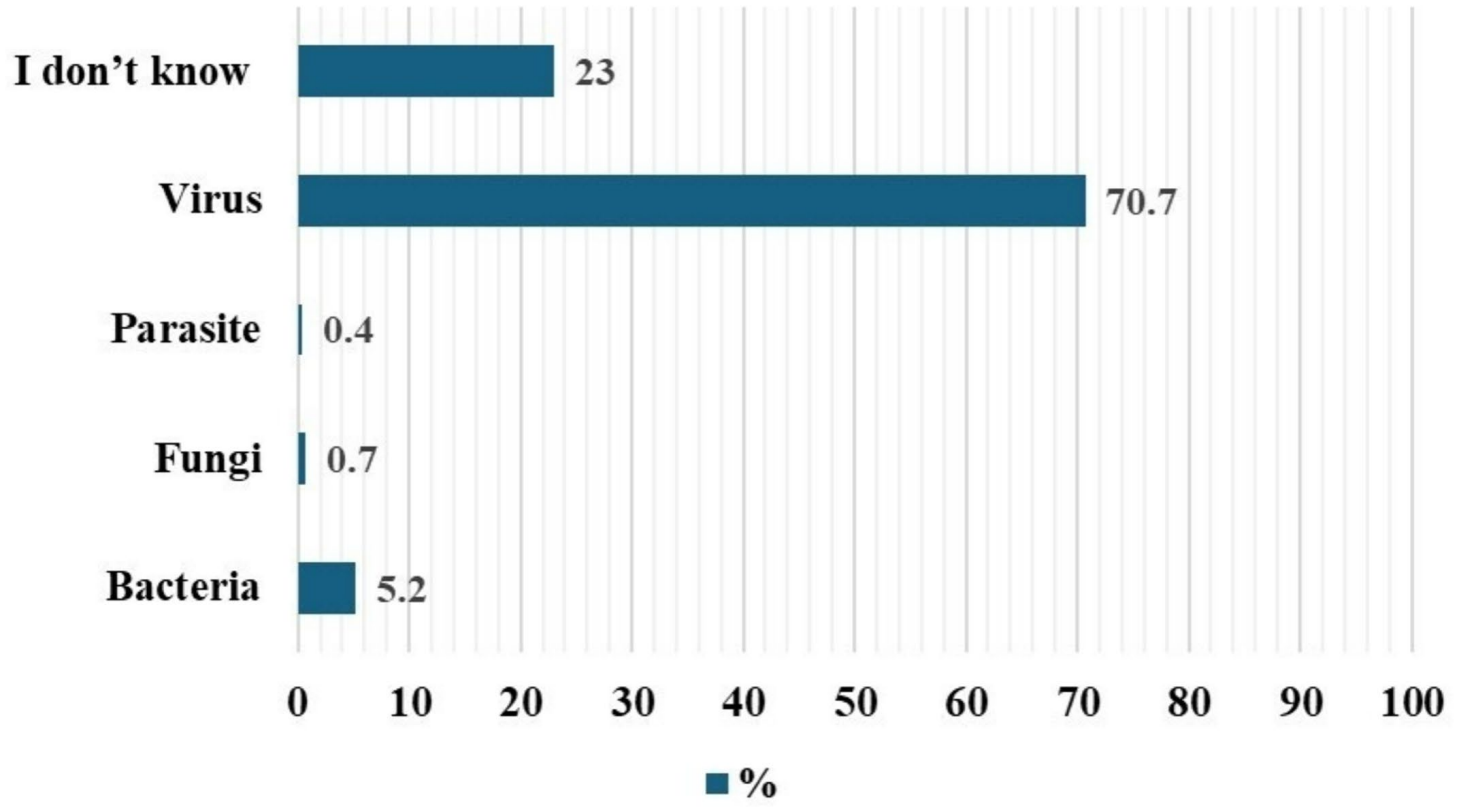
University student perception regarding the cause of Mpox disease.

Regarding etiology, 70.7% correctly identified viruses as the causative agent, while 23.0% were unsure, and a small proportion selected bacteria, fungi, or parasites (Figure 1).

**Figure 2:**
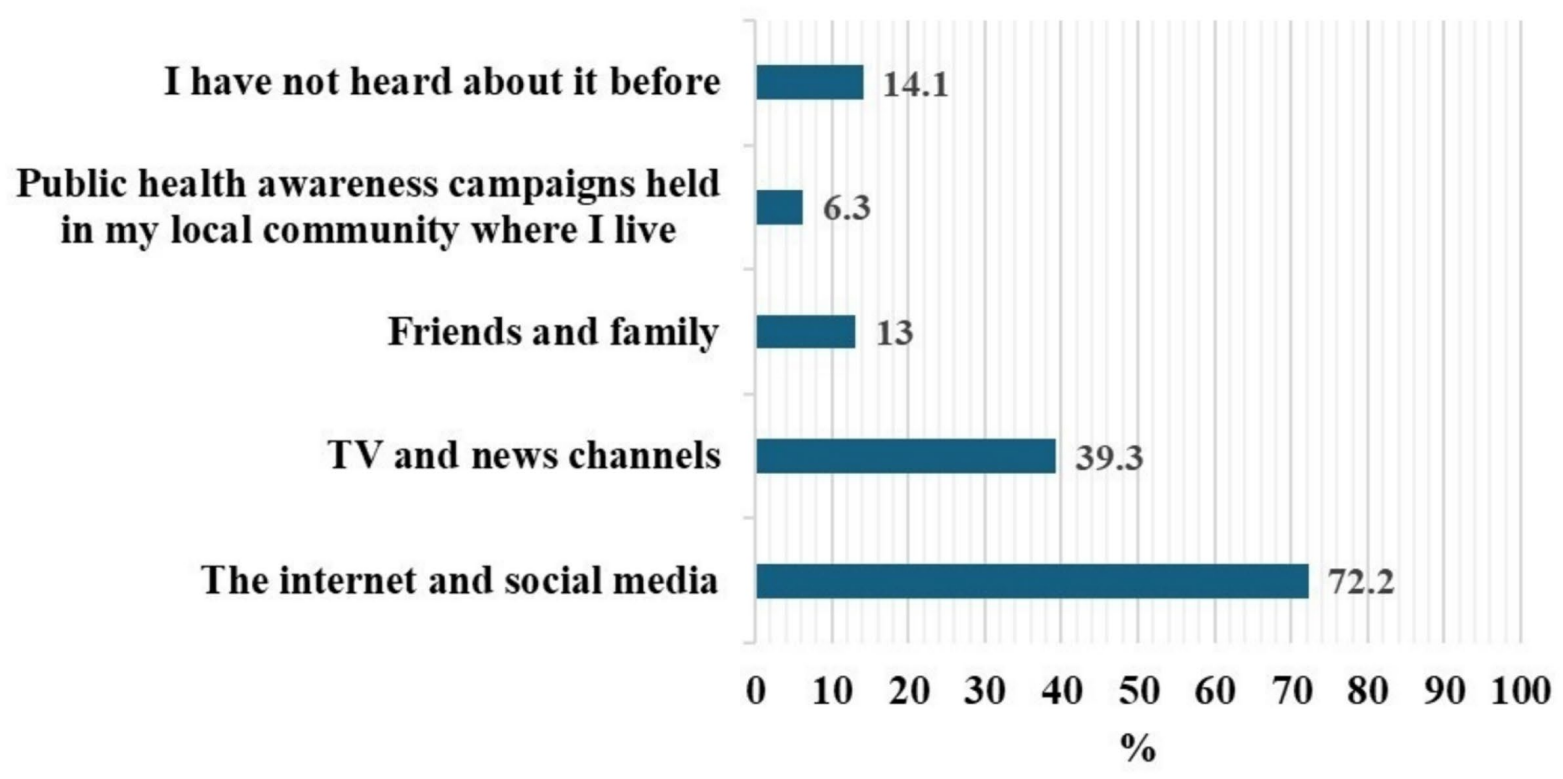
Sources of information about Mpox disease among university students (*n* = 810).

The most commonly reported sources of information were internet/social media (72.2%), followed by television/news channels (39.3%), friends and family (13.0%), and public health campaigns (6.3%). Notably, 14.1% of students reported they had not heard about Mpox before the survey (Figure 2).

### 3.3 Clinical features and risk perception

**Figure 3:**
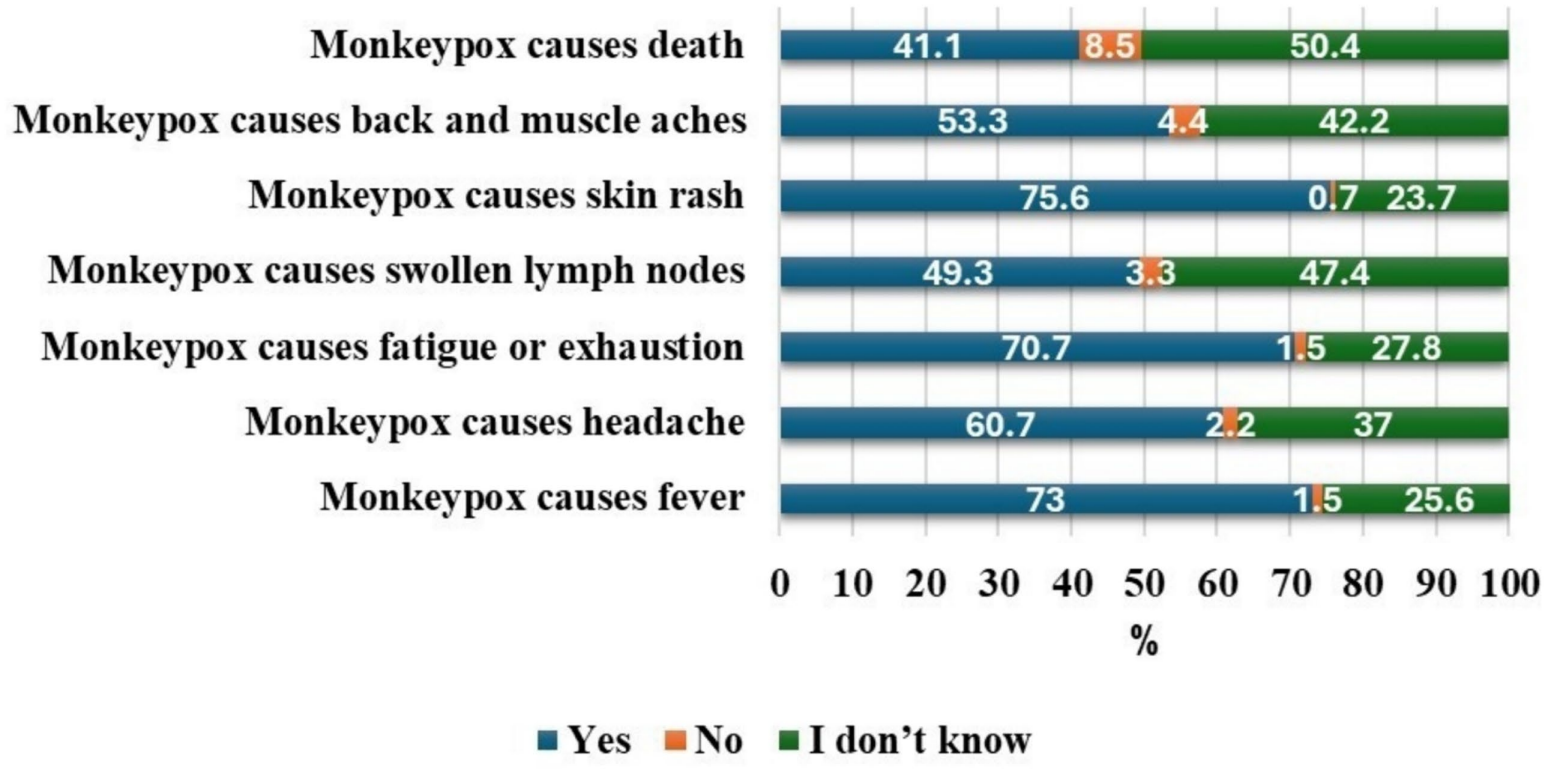
Students’ perception and awareness regarding the clinical features of Mpox. *Percentages may not total 100% due to rounding.

Students identified key Mpox symptoms as follows: rash (75.6%), fever (73.0%), fatigue (70.7%), headache (60.7%), back/muscle aches (53.3%), and lymphadenopathy (49.3%). Regarding severity, 41.1% believed Mpox could result in death, 8.5% disagreed, and 50.4% were unsure (Figure 3).

### 3.4 Attitudes and preventive practices

Preventive behaviors and vaccination attitudes varied (Table 3). Handwashing was widely endorsed (61.7%), while mask-wearing was reported by 59.3%. Willingness to receive a vaccine was moderate overall (50.4%), with higher support for mandatory vaccination among healthcare workers (64.4%) compared with the general population (37.3%).

**Table 3:** Students’ perceptions, attitudes, and practices regarding Mpox prevention and vaccination (*n* = 810).

Questions	Strongly agree n (%)	Agree n (%)	Neutral n (%)	Disagree n (%)	Strongly disagree n (%)
I am worried that monkeypox could cause a global pandemic like COVID-19	21 (2.6)	180 (22.2)	285 (35.2)	324 (40.0)	0 (0)
Since the re-emergence of monkeypox, I have started taking more precautions	225 (27.8)	0 (0)	57 (7.0)	528 (65.2)	0 (0)
I am afraid to travel to any country due to monkeypox	231 (28.5)	0 (0)	78 (9.6)	501 (61.9)	0 (0)
Washing hands is an effective method for preventing monkeypox infection	500 (61.7)	28 (3.5)	168 (20.7)	114 (14.1)	0 (0)
Wearing a mask in public spaces is an effective method for preventing monkeypox infection	86 (10.6)	400 (49.4)	168 (20.7)	91 (11.2)	65 (8.0)
If a monkeypox vaccine becomes available, I am willing to take it	136 (16.8)	272 (33.6)	108 (13.3)	196 (24.2)	98 (12.1)
Monkeypox vaccination should be compulsory for the general population	49 (6.1)	302 (37.3)	99 (12.2)	343 (42.3)	17 (2.1)
Monkeypox vaccination should be compulsory for healthcare workers	22 (2.7)	500 (61.7)	147 (18.2)	141 (17.4)	0 (0)
The benefits of the monkeypox vaccine outweigh its possible side effects	0 (0)	249 (30.7)	369 (45.6)	192 (23.7)	0 (0)
The monkeypox vaccine is unnecessary because the immune system alone is sufficient for protection	68 (8.4)	100 (12.3)	351 (43.3)	202 (24.9)	89 (11.0)

*Percentages may not total 100% due to rounding. Responses were measured using a 5-point Likert scale: 1 = Strongly Agree, 2 = Agree, 3 = Neutral, 4 = Disagree, 5 = Strongly Disagree. Frequencies presented in the table represent raw responses, while numerically coded values were used for statistical analyses.

**Figure 4:**
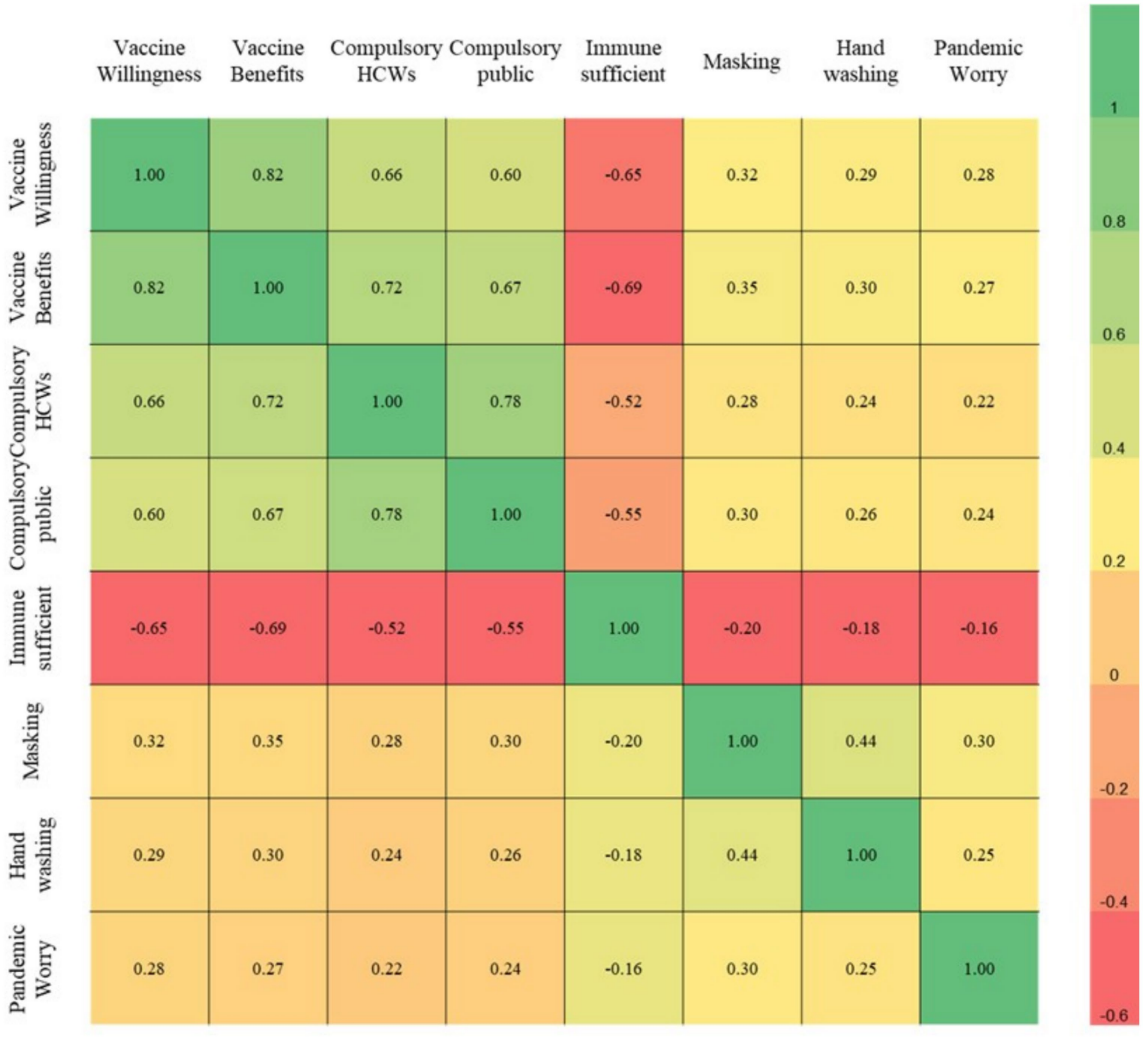
Spearman’s rank-order correlation heatmap of students’ perceptions, attitudes, and practices regarding Mpox prevention and vaccination.

Correlation analyses indicated strong positive associations between vaccine willingness and perceived benefit (*ρ* = 0.82, *p* < 0.001), and between support for mandatory vaccination in healthcare workers and the general population (ρ = 0.78, *p* < 0.001). Precautionary practices were weakly correlated with pandemic-related concern (ρ = 0.25–0.30; Figure 4).

### 3.5 Students’ knowledge and awareness by demographics

Mpox knowledge was summarized using a 20-item composite score (range: 0–20), categorized as poor (0–9), moderate (10–14), or good (15–20), and this categorized outcome was used in regression analyses. Basic awareness (having heard of Mpox) was higher among younger students (18–20 years), while overall composite knowledge scores (regression-based) were higher among older students (≥25 years; OR = 1.91, *p* = 0.047; Table 4). Females demonstrated both higher basic awareness and composite knowledge compared with males (*p* < 0.001; Table 5). Knowledge and perceptions differed across colleges (*p*-values range 0.004–0.025), with medical students showing consistently higher scores. Across colleges, medical students consistently showed higher knowledge scores compared with non-medical programs. Patterns of vaccine willingness were broadly similar, with slightly higher support in healthcare-related programs; extreme percentages in small subgroups (e.g., Applied Medical Sciences, Dentistry, and Education) are reported without emphasis. Significant differences in knowledge and perceptions across colleges were observed for: awareness of the WHO declaration (*p* = 0.025), perceptions of susceptibility (*p* < 0.001), recognition of Mpox as dangerous (*p* < 0.001), transmission via human contact, sexual contact, and contaminated objects (all *p* < 0.001), impact on daily activities (*p* = 0.011), knowledge of available treatments (*p* < 0.001), vaccine availability (*p* < 0.001), expectations of a pandemic (*p* = 0.010), and causative agent (*p* = 0.004; Table 6).

**Table 4:** Knowledge and perceptions regarding Mpox across different age groups (*n* = 810).

Questions	Responses	Age groups (years) n (%)			*p*value
		18–20	21–25	> 25	
Have you ever heard of monkeypox?	Yes	417 (51.5)	243 (30)	36 (4.4)	0.001*
	No	39 (4.8)	72 (8.9)	3 (0.4)	
Have you ever heard that the WHO declared the global monkeypox outbreak a public health emergency of international concern on July 23, 2022?	Yes	285 (35.2)	147 (18.1)	21 (2.6)	< 0.001*
	No	171 (21.1)	168 (20.7)	18 (2.2)	
People are generally susceptible to monkeypox	Yes	273 (33.7)	147 (18.1)	15 (1.9)	< 0.001*
	No	33 (4.1)	42 (5.2)	0 (0.0)	
	I do not know	150 (18.5)	126 (15.6)	24 (3.0)	
Monkeypox is a dangerous infectious disease	Yes	351 (43.3)	189 (23.3)	24 (3.0)	< 0.001*
	No	12 (1.5)	24 (3.0)	0 (0.0)	
	I do not know	93 (11.5)	102 (12.6)	15 (1.9)	
Monkeypox virus can be transmitted through physical contact with other humans	Yes	348 (43.0)	195 (24.1)	27 (3.3)	< 0.001*
	No	15 (1.9)	15 (1.9)	3 (0.4)	
	I do not know	93 (11.5)	105 (13.0)	9 (1.1)	
Monkeypox Virus can be transmitted through physical contact with infected animals	Yes	324 (40.0)	195 (24.1)	27 (3.3)	0.061
	No	6 (0.7)	9 (1.1)	0 (0.0)	
	I do not know	126 (15.6)	111 (13.7)	12 (1.5)	
Monkeypox virus can be transmitted through sexual intercourse	Yes	228 (28.1)	132 (16.3)	6 (0.7)	< 0.001*
	No	21 (2.6)	27 (3.3)	3 (0.4)	
	I do not know	207 (25.6)	156 (19.3)	30 (3.7)	
Monkeypox virus can be transmitted through sharing contaminated sheets, clothes or needles	Yes	261 (32.2)	126 (15.6)	9 (1.1)	< 0.001*
	No	15 (1.9)	33 (4.1)	3 (0.4)	
	I do not know	180 (22.2)	156 (19.3)	27 (3.3)	
Monkeypox can survive for several days on contaminated surfaces	Yes	183 (22.6)	78 (9.6)	6 (0.7)	< 0.001*
	No	21 (2.6)	36 (4.4)	3 (0.4)	
	I do not know	252 (31.1)	201 (24.8)	30 (3.7)	
Do you think monkeypox affects daily activity?	Yes	210 (25.9)	141 (17.4)	12 (1.5)	0.001*
	No	99 (12.2)	39 (4.8)	6 (0.7)	
	I do not know	147 (18.1)	135 (16.7)	21 (2.6)	
There are currently no specific treatments for monkeypox	Yes	141 (17.4)	87 (10.7)	6 (0.7)	0.129
	No	48 (5.9)	42 (5.2)	3 (0.4)	
	I do not know	267 (33.0)	186 (23.0)	30 (3.7)	
There is a vaccine that protects against monkeypox	Yes	156 (19.3)	84 (10.4)	3 (0.4)	< 0.001*
	No	57 (7.0)	42 (5.2)	0 (0.0)	
	I do not know	243 (30.0)	189 (23.3)	36 (4.4)	
Do you expect monkeypox to cause a pandemic like COVID-19?	Yes	165 (20.4)	84 (10.4)	6 (0.7)	0.004*
	No	135 (16.7)	96 (11.9)	18 (2.2)	
	I do not know	156 (19.3)	135 (16.7)	15 (1.9)	
Cause of monkeypox disease	Virus	336 (41.5)	210 (25.9)	27 (3.3)	<0.001*
	Bacteria	15 (1.9)	18 (2.2)	9 (1.1)	
	Fungi	6 (0.7)	0 (0.0)	0 (0.0)	
	Parasite	3 (0.4)	0 (0.0)	0 (0.0)	
	I do not know	96 (11.9)	87 (10.7)	3 (0.4)	

N, number of participants; %, percentage of total participants; *p ≤ 0.05 indicates statistical significance.

**Table 5:** Relationship between sex and Mpox-related knowledge and perception (*n* = 810).

Questions	Responses	Sex n (%)		*p* value
		Male	Female	
Have you ever heard of monkeypox?	Yes	240 (29.6)	456 (56.3)	< 0.001*
	No	66 (8.1)	48 (5.9)	
Have you ever heard that the WHO declared the global monkeypox outbreak a public health emergency of international concern on July 23, 2022?	Yes	141 (17.4)	333 (41.1)	< 0.001*
	No	165 (20.4)	171 (21.1)	
People are generally susceptible to monkeypox	Yes	147 (18.1)	288 (35.6)	0.039*
	No	30 (3.7)	45 (5.6)	
	I do not know	129 (15.9)	171 (21.1)	
Monkeypox is a dangerous infectious disease	Yes	186 (23.0)	378 (46.7)	< 0.001*
	No	24 (3.0)	12 (1.5)	
	I do not know	96 (11.9)	114 (14.1)	
Monkeypox virus can be transmitted through physical contact with other humans	Yes	180 (22.2)	390 (48.1)	< 0.001*
	No	18 (2.2)	15 (1.9)	
	I do not know	108 (13.3)	99 (12.2)	
Monkeypox Virus can be transmitted through physical contact with infected animals	Yes	183 (22.6)	363 (44.8)	< 0.001*
	No	3 (0.4)	12 (1.5)	
	I do not know	120 (14.8)	129 (15.9)	
Monkeypox virus can be transmitted through sexual intercourse	Yes	114 (14.1)	252 (31.1)	0.002*
	No	21 (2.6)	30 (3.7)	
	I do not know	171 (21.1)	222 (27.4)	
Monkeypox virus can be transmitted through sharing contaminated sheets, clothes or needles	Yes	120 (14.8)	276 (34.1)	< 0.001*
	No	27 (3.3)	24 (3.0)	
	I do not know	159 (19.6)	204 (25.2)	
Monkeypox can survive for several days on contaminated surfaces	Yes	69 (8.5)	198 (24.4)	0.001*
	No	36 (4.4)	24 (3.0)	
	I do not know	201 (24.8)	282 (34.8)	
Do you think monkeypox affects daily activity?	Yes	93 (11.5)	270 (33.3)	0.001*
	No	78 (9.6)	66 (8.1)	
	I do not know	135 (16.7)	168 (20.7)	
There are currently no specific treatments for monkeypox	Yes	84 (10.4)	150 (18.5)	0.274
	No	42 (5.2)	51 (6.3)	
	I do not know	180 (22.2)	303 (37.4)	
There is a vaccine that protects against monkeypox	Yes	69 (8.5)	174 (21.5)	< 0.001*
	No	36 (4.4)	63 (7.8)	
	I do not know	201 (24.8)	267 (33.0)	
Do you expect monkeypox to cause a pandemic like COVID-19?	Yes	78 (9.6)	177 (21.9)	0.002*
	No	114 (14.1)	192 (23.7)	
	I do not know	114 (14.1)	135 (16.7)	
Cause of monkeypox disease	Virus	189 (23.3)	384 (47.4)	< 0.001*
	Bacteria	24 (3.0)	18 (2.2)	
	Fungi	0 (0.0)	6 (0.7)	
	Parasite	0 (0.0)	3 (0.4)	
	I do not know	93 (11.5)	93 (11.5)	

N, number of participants; %, percentage of total participants; *p*-values were calculated using chi-square test to compare responses between males and females; *Indicates statistically significant difference at *p* ≤ 0.05.

**Table 6:** College-wise comparison of Mpox knowledge, awareness, and perceptions among students (*n* = 810).

Questions	Responses	College n (%)						*p*value
		Medicine	Pharmacy	Nursing	Applied medical sciences	Dentistry	Non-medical	
Have you ever heard of monkey pox?	Yes	492 (60.7)	45 (5.6)	18 (2.2)	51 (6.3)	18 (2.2)	72 (8.9)	0.015*
	No	96 (11.9)	3 (0.4)	0 (0.0)	3 (0.4)	0 (0.0)	12 (1.5)	
Have you ever heard that the WHO declared the global monkeypox outbreak a public health emergency of international concern on July 23, 2022?	Yes	325 (40.1)	30 (3.7)	12 (1.5)	39 (4.8)	15 (1.9)	53 (6.5)	0.025*
	No	263 (32.5)	18 (2.2)	6 (0.7)	15 (1.9)	3 (0.4)	31 (3.8)	
People are generally susceptible to monkeypox	Yes	296 (36.5)	36 (4.4)	18 (2.2)	33 (4.1)	12 (1.5)	40 (4.9)	<0.001*
	No	56 (6.9)	3 (0.4)	0 (0.0)	6 (0.7)	0 (0.0)	10 (1.2)	
	I do not know	236 (29.1)	9 (1.1)	0 (0.0)	15 (1.9)	6 (0.7)	34 (4.2)	
Monkeypox is a dangerous infectious disease	Yes	405 (50)	42 (5.2)	18 (2.2)	36 (4.4)	18 (2.2)	51 (6.3)	<0.001*
	No	30 (3.7)	0 (0.0)	0 (0.0)	3 (0.4)	0 (0.0)	3 (0.4)	
	I do not know	156 (19.3)	6 (0.7)	0 (0.0)	15 (1.9)	0 (0.0)	30 (3.7)	
Monkeypox virus can be transmitted through physical contact with other humans	Yes	421 (52)	42 (5.2)	12 (1.5)	30 (3.7)	18 (2.2)	47 (5.8)	<0.001*
	No	27 (3.3)	3 (0.4)	3 (0.4)	0 (0.0)	0 (0.0)	0 (0.0)	
	I do not know	140 (17.3)	3 (0.4)	3 (0.4)	24 (3)	0 (0.0)	37 (4.6)	
Monkeypox Virus can be transmitted through physical contact with infected animals	Yes	418 (51.6)	33 (4.1)	12 (1.5)	30 (3.7)	15 (1.9)	49 (6.0)	0.052
	No	11 (1.4)	0 (0.0)	0 (0.0)	0 (0.0)	0 (0.0)	4 (0.5)	
	I do not know	159 (19.6)	15 (1.9)	6 (0.7)	24 (3)	3 (0.4)	31 (3.8)	
Monkeypox virus can be transmitted through sexual intercourse	Yes	250 (30.9)	27 (3.3)	9 (1.1)	33 (4.1)	15 (1.9)	32 (4)	<0.001*
	No	39 (4.8)	0 (0.0)	9 (1.1)	0 (0.0)	0 (0.0)	3 (0.4)	
	I do not know	299 (36.9)	21 (2.6)	0 (0.0)	21 (2.6)	3 (0.4)	49 (6.0)	
Monkeypox virus can be transmitted through sharing contaminated sheets, clothes or needles	Yes	277 (34.2)	30 (3.7)	15 (1.9)	21 (2.6)	15 (1.9)	38 (4.7)	<0.001*
	No	45 (5.6)	0 (0.0)	3 (0.4)	3 (0.4)	0 (0.0)	0 (0.0)	
	I do not know	266 (32.8)	18 (2.2)	0 (0.0)	30 (3.7)	3 (0.4)	46 (5.7)	
Monkeypox can survive for several days on contaminated surfaces	Yes	192 (23.7)	21 (2.6)	9 (1.1)	15 (1.9)	6 (0.7)	24 (3)	<0.001*
	No	44 (5.4)	0 (0.0)	6 (0.7)	6 (0.7)	3 (0.4)	1 (0.1)	
	I do not know	352 (43.5)	27 (3.3)	3 (0.4)	33 (4.1)	9 (1.1)	59 (7.3)	
Do you think monkeypox affects daily activity?	Yes	256 (31.6)	18 (2.2)	9 (1.1)	30 (3.7)	6 (0.7)	44 (5.4)	0.011*
	No	236 (29.1)	24 (3)	3 (0.4)	12 (1.5)	6 (0.7)	22 (2.7)	
	I do not know	96 (11.9)	6 (0.7)	6 (0.7)	12 (1.5)	6 (0.7)	18 (2.2)	
There are currently no specific treatments for monkeypox	Yes	157 (19.4)	27 (3.3)	6 (0.7)	18 (2.2)	9 (1.1)	17 (2.1)	<0.001*
	No	71 (8.8)	3 (0.4)	6 (0.7)	6 (0.7)	3 (0.4)	4 (0.5)	
	I do not know	360 (44.4)	18 (2.2)	6 (0.7)	30 (3.7)	6 (0.7)	63 (7.8)	
There is a vaccine that protects against monkeypox	Yes	169 (20.9)	15 (1.9)	12 (1.5)	15 (1.9)	12 (1.5)	20 (2.5)	<0.001*
	No	78 (9.6)	6 (0.7)	0 (0.0)	9 (1.1)	0 (0.0)	6 (0.7)	
	I do not know	341 (42.1)	27 (3.3)	6 (0.7)	30 (3.7)	6 (0.7)	58 (7.2)	
Do you expect monkeypox to cause a pandemic like COVID-19?	Yes	183 (22.6)	18 (2.2)	3 (0.4)	18 (2.2)	6 (0.7)	27 (3.3)	0.010*
	No	211 (26.0)	15 (1.9)	15 (1.9)	24 (3)	9 (1.1)	32 (4)	
	I do not know	194 (24)	15 (1.9)	0 (0.0)	12 (1.5)	3 (0.4)	25 (3.1)	
Cause of monkeypox disease	Virus	402 (49.6)	39 (4.8)	18 (2.2)	42 (5.2)	18 (2.2)	54 (6.7)	0.004*
	Bacteria	42 (5.2)	0 (0.0)	0 (0.0)	0 (0.0)	0 (0.0)	0 (0.0)	
	Fungi	6 (0.7)	0 (0.0)	0 (0.0)	0 (0.0)	0 (0.0)	0 (0.0)	
	Parasite	3 (0.4)	0 (0.0)	0 (0.0)	0 (0.0)	0 (0.0)	0 (0.0)	
	I do not know	135 (16.7)	9 (1.1)	0 (0.0)	12 (1.5)	0 (0.0)	30 (3.7)	

N, number of participants; %, percentage of total participants; *p*-values were calculated using chi-square test to compare responses across college; * indicates statistically significant difference (*p* ≤ 0.05). Non-medical includes participants from colleges other than Medicine, Pharmacy, Nursing, Applied Medical Sciences, and Dentistry.

### 3.6 Determinants of knowledge and information sources

**Figure 5:**
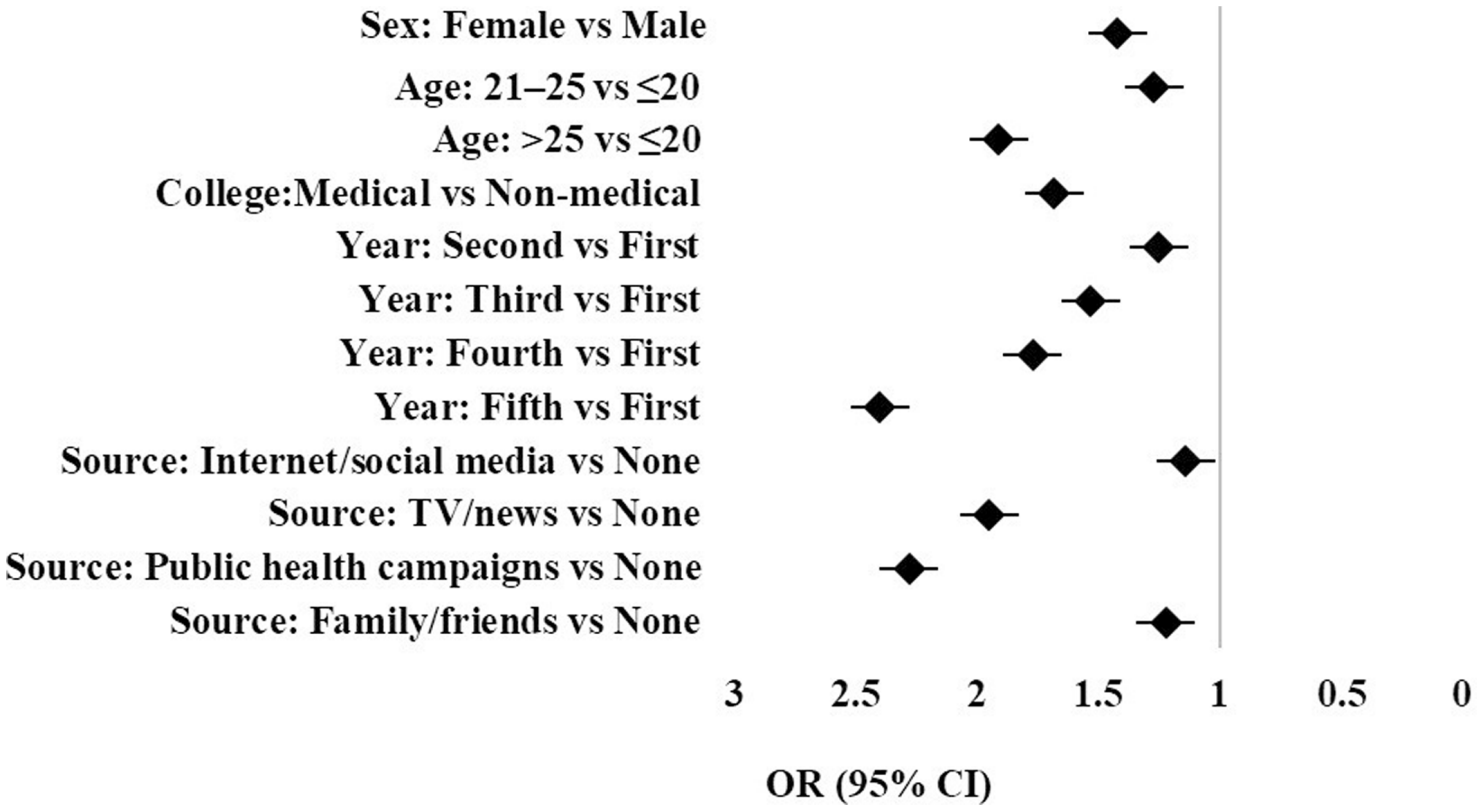
Forest plot curve for significant factors associated with the good and moderate knowledge about Mpox infection.

**Figure 6:**
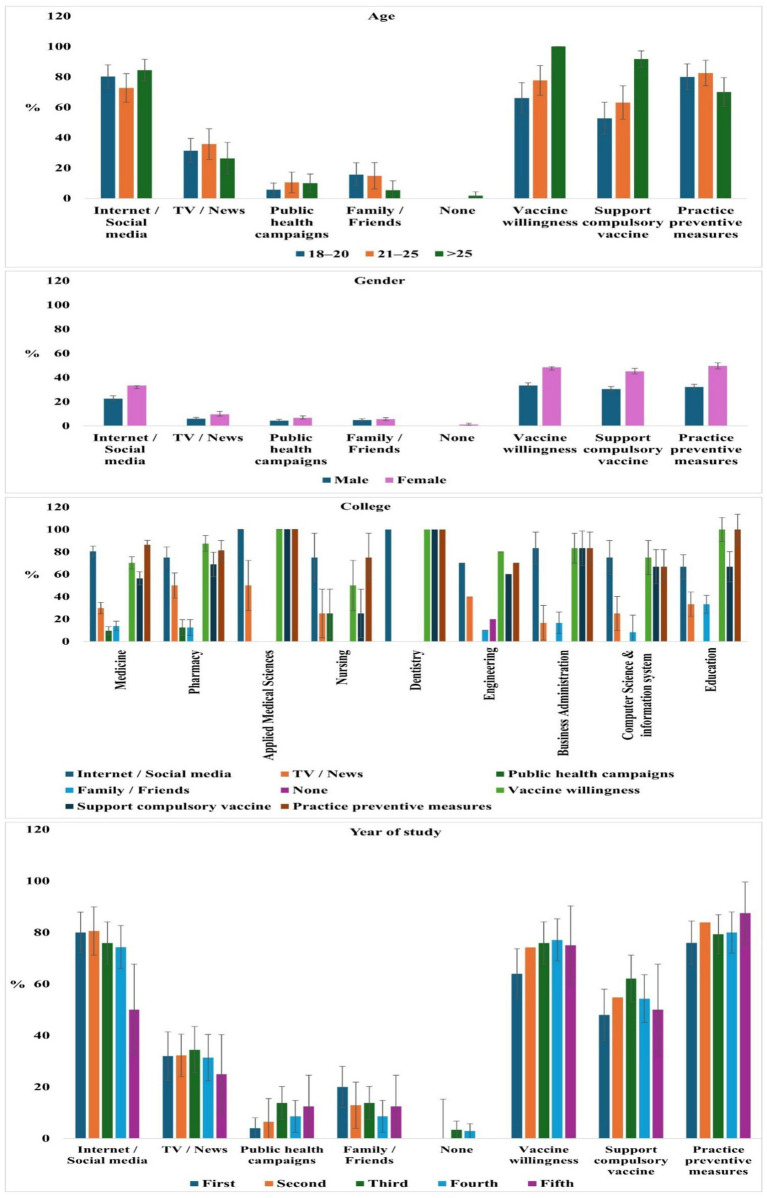
Distribution of information sources and attitudes toward Mpox vaccination and preventive measures across demographic groups. Bars represent percentages of participants; error bars indicate the standard error (SE) for each group.

Multivariable logistic regression identified female gender (OR = 1.42, *p* = 0.038), age ≥25 years (OR = 1.91, *p* = 0.047), enrollment in medical programs (OR = 1.68, *p* = 0.008), and information from TV/news (OR = 1.95, *p* = 0.008) or public health campaigns (OR = 2.28, *p* = 0.006) as significant predictors of moderate/good knowledge (Figure 5). Internet/social media and family/friends were the most common sources but were not significantly associated with higher knowledge scores. Vaccine willingness and preventive practices were generally positive across demographics (Figure 6). Patterns across small subgroups are noted but not highlighted to avoid overemphasis.

## 4 Discussion

This study examined Mpox-related knowledge, perceptions, attitudes, and preventive practices among undergraduate students in Riyadh, Saudi Arabia. Overall, general awareness of Mpox was high; however, substantial gaps persisted in detailed knowledge regarding transmission routes, clinical features, vaccine availability, and treatment options. This pattern reflects a distinction between surface awareness and comprehensive understanding, a recurring theme in emerging infectious disease preparedness. Across the Middle East and South Asia, studies consistently report widespread awareness coupled with limited depth of understanding, particularly regarding transmission routes, preventive strategies, and vaccination (10–15). For instance, studies from Saudi Arabia, Jordan, Iran, Pakistan, and the United States show that while most students had heard of Mpox, detailed knowledge remained inadequate. Collectively, these findings suggest that mere exposure to information is insufficient without structured, context-specific health education.

Similar patterns have been reported in other university-based studies in Saudi Arabia. A cross-sectional study among pharmacy students at Jazan University found that only 32% of participants demonstrated a high level of knowledge and 44.5% showed a positive attitude toward Mpox, indicating generally limited awareness even among health-related disciplines (16). Likewise, another study among medical students reported that 84% of participants had poor knowledge of Mpox, with substantial gaps in understanding symptoms, transmission routes, and vaccine availability (17). In both studies, social media was identified as the primary source of information, while relatively few students reported receiving structured information about Mpox through formal medical education. Despite these knowledge gaps, many students expressed willingness to adopt preventive measures and receive vaccination if available, suggesting that positive attitudes toward prevention may exist even when knowledge remains incomplete. Together, these findings support the results of the present study and highlight the importance of strengthening university-based health education and awareness programs to improve understanding of emerging infectious diseases among students.

In this study, although internet and social media platforms were the most frequently cited sources, traditional media (TV/news) and structured public health campaigns were independently associated with higher knowledge scores. Professionally curated sources likely convey more accurate and actionable health information than unregulated digital content (10, 11, 13, 18–20). In a Saudi context characterized by high social media penetration, this indicates that public health authorities should actively engage digital platforms while ensuring message accuracy and credibility. Superficial exposure through social media alone may explain the high basic awareness among younger students, without corresponding depth of knowledge.

Moderate vaccine acceptance in this cohort reflects both perceived disease severity and uncertainty regarding vaccine safety and necessity. Greater support for mandatory vaccination among healthcare workers than the general population indicates that students differentiate between professional responsibility and personal risk. Consistent with the Health Belief Model, vaccine willingness was associated with perceived risk (perceived susceptibility) and belief in vaccine benefits (perceived benefits), whereas uncertainty about safety reflected perceived barriers. Targeted messaging addressing these factors is therefore essential for improving vaccine uptake in university populations.

Higher knowledge levels among medical students, females, and students exposed to structured public health messaging are consistent with prior research emphasizing the role of educational background, academic exposure, and access to scientific resources (21–23). Gender differences may reflect higher health information-seeking behavior, risk perception, and engagement with educational content among female students, potentially influenced by sociocultural norms that shape attention to health information. Younger students reported higher basic awareness of Mpox, likely due to rapid exposure via digital media. In contrast, older students had higher odds of moderate or good composite knowledge in multivariable analysis, suggesting that comprehensive understanding develops through cumulative academic experience and repeated exposure to structured health information. This distinction between superficial awareness and consolidated knowledge reconciles the apparent contradiction observed between descriptive and regression analyses and aligns with comparable studies (24–26).

Several limitations should be noted. First, the predominance of medical students (72.6%) is the most significant threat to external validity, likely leading to an overestimation of overall knowledge levels. Second, convenience sampling and voluntary participation may have introduced selection bias. Third, reliance on self-reported data could contribute to information bias. Fourth, formal validation of the questionnaire, including reliability testing such as Cronbach’s alpha, was not conducted, which may limit the precision of the knowledge and attitude measurements. Finally, the study was conducted in Riyadh, limiting generalizability to other regions or non-medical institutions. Despite these constraints, the large sample size and inclusion of multiple academic disciplines provide robust evidence for associations within this university population, offering actionable insights for targeted interventions.

## 5 Conclusion

This study highlights a clear distinction between general awareness and comprehensive knowledge of Mpox among university students in Riyadh, Saudi Arabia. While exposure to information, particularly via digital media was widespread, accurate understanding of transmission, prevention, treatment, and vaccination remains limited. These findings underscore the need for targeted, evidence-based health education programs that prioritize non-medical students, males, and younger adults, integrating both digital platforms and trusted institutional or public health channels. University administrators, educators, and public health authorities should implement structured interventions to close knowledge gaps, promote preventive behaviors, and improve vaccine acceptance, thereby strengthening Mpox preparedness among young adults.

## Data Availability

The original contributions presented in the study are included in the article/supplementary material, further inquiries can be directed to the corresponding author.
